# Mobile App Prototype in Older Adults for Postfracture Acute Pain Management: User-Centered Design Approach

**DOI:** 10.2196/37772

**Published:** 2022-10-17

**Authors:** Kevin Tran-Nguyen, Caroline Berger, Roxanne Bennett, Michelle Wall, Suzanne N Morin, Fateme Rajabiyazdi

**Affiliations:** 1 Research Institute of the McGill University Health Centre Montreal, QC Canada; 2 University of Maryland College of Information Studies College Park, MD United States; 3 Department of Systems and Computer Engineering Carleton University Ottawa, ON Canada

**Keywords:** older adults, mobile app, skeletal fracture, usability, patient-centered, human-centered design, digital health, eHealth, mobile health, mHealth, acute pain self-management, mobile phone

## Abstract

**Background:**

Postfracture acute pain is often inadequately managed in older adults. Mobile health (mHealth) technologies can offer opportunities for self-management of pain; however, insufficient apps exist for acute pain management after a fracture, and none are designed for an older adult population.

**Objective:**

This study aims to design, develop, and evaluate an mHealth app prototype using a human-centered design approach to support older adults in the self-management of postfracture acute pain.

**Methods:**

This study used a multidisciplinary and user-centered design approach. Overall, 7 stakeholders (ie, 1 clinician-researcher specialized in internal medicine, 2 user experience designers, 1 computer science researcher, 1 clinical research assistant researcher, and 2 pharmacists) from the project team, together with 355 external stakeholders, were involved throughout our user-centered development process that included surveys, requirement elicitation, participatory design workshops, mobile app design and development, mobile app content development, and usability testing. We completed this study in 3 phases. We analyzed data from prior surveys administered to 305 members of the Canadian Osteoporosis Patient Network and 34 health care professionals to identify requirements for designing a low-fidelity prototype. Next, we facilitated 4 participatory design workshops with 6 participants for feedback on content, presentation, and interaction with our proposed low-fidelity prototype. After analyzing the collected data using thematic analysis, we designed a medium-fidelity prototype. Finally, to evaluate our medium-fidelity prototype, we conducted usability tests with 10 participants. The results informed the design of our high-fidelity prototype. Throughout all the phases of this development study, we incorporated inputs from health professionals to ensure the accuracy and validity of the medical content in our prototypes.

**Results:**

We identified 3 categories of functionalities necessary to include in the design of our initial low-fidelity prototype: the need for support resources, diary entries, and access to educational materials. We then conducted a thematic analysis of the data collected in the design workshops, which revealed 4 themes: feedback on the user interface design and usability, requests for additional functionalities, feedback on medical guides and educational materials, and suggestions for additional medical content. On the basis of these results, we designed a medium-fidelity prototype. All the participants in the usability evaluation tests found the medium-fidelity prototype useful and easy to use. On the basis of the feedback and difficulties experienced by participants, we adjusted our design in preparation for the high-fidelity prototype.

**Conclusions:**

We designed, developed, and evaluated an mHealth app to support older adults in the self-management of pain after a fracture. The participants found our proposed prototype useful for managing acute pain and easy to interact with and navigate. Assessment of the clinical outcomes and long-term effects of our proposed mHealth app will be evaluated in the future.

## Introduction

### Background

The rate of incidence of fractures is increasing globally, particularly in older adults [1]. Fractures are associated with acute pain, loss of autonomy, anxiety, recurrent falls, and, in a significant proportion, transition to chronic pain syndromes [2-4]. Furthermore, 74% of the patients visiting the emergency department, including those who present with at least moderate pain after a fracture or dislocation, are discharged with moderate to severe pain [5].

Multimodal approaches, including the use of medications, restorative therapies, and behavioral and complementary health approaches, are recommended for the management of acute pain conditions, including pain experienced following a skeletal fracture [6,7]. Despite a series of guidelines established by the US Institute of Medicine and the American Pain Society to manage pain [2,3], acute pain is underrecognized and undertreated in older adults both in and out of the hospital setting, leading to negative clinical outcomes [2]. In response to inadequate outpatient acute pain management, the US Department of Health and Human Services has emphasized the need for individualized self-management programs to support older patients in coping with and reducing their pain through pharmacological and nonpharmacological methods [6].

With the increased availability and use of mobile health (mHealth) tools on smartphones that are commercially available to users, there is a growing opportunity to develop mobile apps offering individualized pain management. The types of mHealth mobile apps that have been shown to have common clinical value offer at least one of the following: supporting clinical diagnosis, promoting behavior change and increasing patient adherence with treatment plans, supporting self-management of a condition, or delivering disease-related education [8]. However, systematic and scoping reviews have found that most mHealth apps were developed for chronic pain rather than for acute pain management [9-12]. A recent scoping review focused on mHealth in the context of surgery found that out of 13 studies, only 5 focused on addressing postsurgery acute pain [13]. These 5 studies aimed to reduce postoperative pain in patients by monitoring opioid use [14-16] and encouraging therapeutic adherence via smartphone functionalities such as alarms and accelerometers [17,18]. Among the mobile apps publicly available for iOS and Android devices, options catering specifically to acute pain are limited. A systematic review of commercially available pain management apps recommended 3 apps: Curable, Pathways, and Vivify; however, all of these apps were designed for chronic pain [19]. The systematic review found Achy Penguin to be the only available app in Canada that specifically manages acute pain, but it is designed for young children and does not fulfill the needs of older adults [19]. Previous studies on the use of mHealth to manage pain were promising in improving pain outcomes, but more research is required in this field, as many mHealth apps remain unvalidated by scientific means [12,20-22].

In addition, most of the pain management mHealth apps are designed without the involvement of health care providers [12] and older adults, resulting in apps that are ill-suited for an older audience [9,22]. Thus, there is a gap in the availability of innovative evidence-based mHealth tools and solutions to support older patients in the management of their acute pain once they leave the hospital.

### Objectives

This study aimed to design, develop, and evaluate an mHealth app prototype to support older adults in the self-management of postfracture acute pain using a human-centered design (HCD) approach, which involves focusing on understanding the context of use, needs, and problems of the end users to develop the technological solution [23,24]. The main focus of this app is on medication management and adherence with support for other pain management needs such as educational materials and external available resources. The novelty of our work lies in the design and development process of the mHealth app, as we established evidence-based design requirements for our prototype and included older adults and health care professionals in the process.

## Methods

### Ethics Approval

Approval was obtained from the research ethics board of the Research Institute of the McGill University Health Centre (approval number 2021-7611), and all participants provided informed consent.

### Overview

We reviewed the literature and conducted an informal competitive analysis to identify mobile apps available in the literature and on the market to manage postfracture acute pain. We did not find any apps that were deemed suitable and clinically valid for older adults to manage their pain after a fracture. Thus, we aimed to develop a high-fidelity mHealth prototype app. We used an HCD approach to ensure that the end product was effective and efficient for the target users [25].

**Figure 1:**
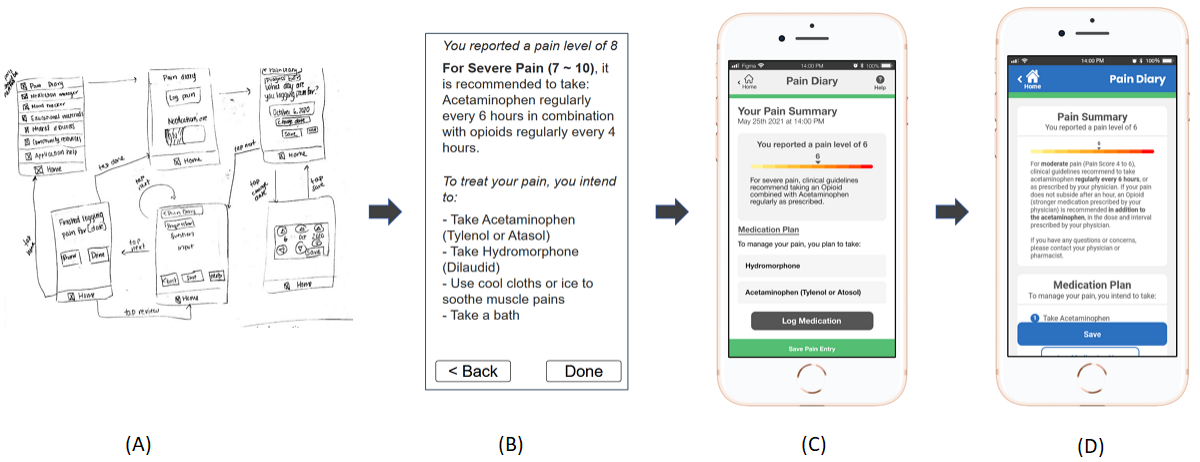
The iterations of our mobile health app from (A) sketches, (B) low-fidelity prototype, (C) medium-fidelity interactive prototype, and (D) high-fidelity interactive prototype. The pages shown below display the logging pain functionality.

First, we identified the design requirements for a low-fidelity prototype using the results from prior surveys of members of the Canadian Osteoporosis Patient Network and clinicians. Next, we facilitated a series of participatory design workshops with older adults who had experienced a fracture and iteratively developed a medium-fidelity prototype. To find areas for improvement and gather evidence on the usability of our medium-fidelity prototype, we conducted usability tests, a method for hunting design and interaction problems in an interface [24]. Finally, we designed a high-fidelity prototype based on the results of the usability tests (Figure 1). In addition, throughout the low- and medium-fidelity prototype phases of the study, we validated the content of our app through one-on-one discussions with pharmacists in our network.

### Phase 1—Initial Design Requirements

#### Overview

We used the information gathered through previous surveys administered by our research team with 305 members of the Canadian Osteoporosis Patient Network and 34 health care professionals (physicians, nurses, physiotherapists, and pharmacists). We used the results of the surveys to gather data from a large population to determine the content and functionalities needed when developing an mHealth app for acute pain self-management. The question asked in the survey to both groups was as follows: What do you believe to be the most important (1) content to include and (2) the functionalities to have on a mHealth app to empower adults aged >60 years to manage acute pain at home after discharge from the emergency department, following management of a skeletal fracture?

#### Data Analysis

The results of the surveys were analyzed to identify design requirements, high-level functionalities, and use cases by means of hierarchical task analysis specifically for the purpose of developing our prototype [26]. In addition, we identified a list of accessibility design guidelines for older adults from the literature [27-30]. Examples of accessibility guidelines that we considered in our design included using large font sizes, high color contrasts, large buttons, simple gestures, consistent layouts, and flattened menu structures. In addition to these accessibility guidelines, we opted for a hub-and-spoke navigation pattern in which users have to backtrack to the home page to access another part of the app, as prior studies have demonstrated that this pattern is easy to navigate for older adults [31,32].

We discussed and iterated sketches of the app within our multidisciplinary team, which included a clinician-researcher specialized in internal medicine, 2 user experience designers, a computer science researcher, and a clinical research assistant. Once we finalized the initial design of our app, we converted the sketches to a digital low-fidelity prototype using Axure (Axure Software Solutions Inc).

### Phase 2—Participatory Design Workshops

#### Recruitment

In total, 6 older adults from Canada were recruited to take part in 4 participatory design workshops. Inclusion criteria for the workshop participants were as follows: they must (1) be aged ≥50 years; (2) have sustained at least one skeletal fracture after the age of 40 years; (3) be able to communicate in English; and (4) have access to the internet and own a desktop computer or laptop computer with a camera and microphone.

#### Procedure

Participatory design is a method that empowers users to become co-designers, inviting them to actively participate in the design process [33,34]. As such, we decided to conduct participatory design workshops so that our targeted end users could directly influence the design. The objective of these design workshops was 2-fold: to obtain feedback on the digital low-fidelity prototype and to uncover unanticipated requirements. We obtained informed consent and demographic information before commencing the participatory design workshops. We facilitated 4 workshops with the same group of participants from March 2021 to July 2021. We conducted the workshops remotely over Zoom (Zoom Video Communications) and audio and video recorded the workshops. Three members of the research team were present during the workshops. One moderated the session; one assisted the moderator in answering questions; and one observed and took notes, occasionally asking confirmatory questions. The participants were shown the prototype through Zoom’s screen-sharing functionality during the workshops. The aim of the first 3 workshops was to seek feedback and suggestions on different parts of the prototype. Upon analyzing the data gathered in the first 3 design workshops, we designed an interactive medium-fidelity prototype, ensuring that all issues raised during the first 3 workshops were addressed. Finally, we demonstrated the medium-fidelity prototype as a whole in the fourth workshop for a final round of feedback.

#### Data Analysis

Two members of our research team were responsible for the thematic analysis following the steps proposed by Braun and Clarke [35]. The goal of this analysis was to summarize the feedback on our prototype and uncover additional user needs that were missed in phase 1 of the study. In the first step, the analysts read the transcripts from the first 3 design workshops and took notes. In the second step, they reviewed the transcripts and generated initial codes. The codes were then compared for agreement and subsequently applied to the fourth design workshop transcript. Our analysts used Quirkos (Quirkos Software) to facilitate the coding process. In the third step, they generated the initial themes, which were then reviewed in the fourth step. In the fifth step, another member of the team contributed inputs to further refine the themes. Finally, in sixth step, we as a group chose representative quotes for each theme and summarized our findings.

### Phase 3—Usability Testing

#### Recruitment

We recruited a convenience sample of 10 older adults from the Greater Montreal Area (Quebec, Canada) to participate in usability tests. Inclusion criteria were as follows: the participants must be (1) aged ≥50 years, (2) have sustained at least one skeletal fracture after the age of 40 years, (3) be able to communicate in English, and (4) have access to the internet and own a desktop computer or laptop computer with a camera and microphone. These participants had not participated in the second phase of our study and did not have any prior knowledge of our app.

#### Procedure

To gain information about users’ behaviors while naturally using the product, we decided to conduct observational studies monitoring the use of our app by the users [36]. Following the guidelines in the literature [37], we used an iterative approach for our usability tests by dividing them into batches. Three participants did the initial test. We then addressed the most glaring usability issues and tested the app again with 4 participants. Finally, we tested the app with 3 additional participants. Because of the iterative nature of our prototype, we did not report quantitative data, such as error frequency. We collected informed consent and demographic information before the usability testing sessions. The same person moderated testing sessions remotely via Zoom, and we audio and video recorded the sessions. The moderator sent a secure link over the Zoom chat and instructed the participants to open the link to the medium-fidelity prototype in their browser and share their screen. Studies have demonstrated that using emulators is an acceptable method of mobile usability testing [38]; thus, we used a mobile device emulator on a desktop to test the mHealth app prototype. We chose to use an emulator for ease of use, as most participants were more familiar with using Zoom on their computers than on their mobile phones. The moderator asked the participants to use the different functionalities within the app while thinking back about their latest fracture experience. For example, “Think back to the time you broke one of your bone(s). Let’s say you’ve just taken two tablets of Tylenol for your pain. How would you keep track of your medication intake using the application?”. They were also instructed to think aloud using a concurrent think-aloud approach [39]. Throughout the usability test, the moderator took notes on any difficulties the participants experienced. At the end of the usability test, the participants answered open-ended questions about their overall experience with the medium-fidelity prototype. The usability test script is presented in Multimedia Appendix 1.

#### Data Analysis

We transcribed the audio recordings of the usability test sessions to uncover any usability problems that may have been noted during the sessions. We analyzed and classified the errors that the participants made while performing each task and reviewed the answers to the open-ended questions.

## Results

### Phase 1—Initial Design Requirements

**Figure 2:**
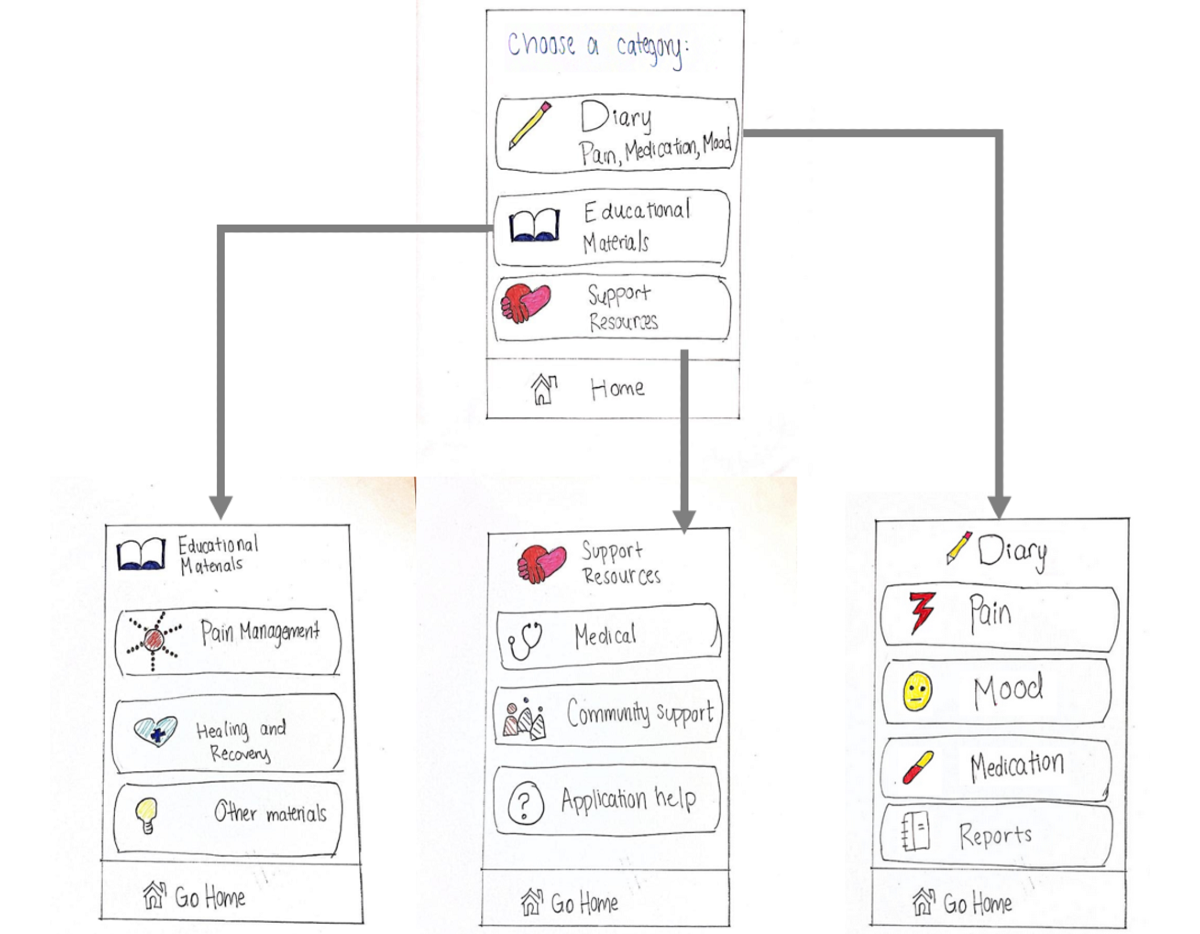
Sketches of the home page and three categories of functionality pages: educational materials, support resources, and diary.

Surveys were conducted with 305 members of the Canadian Osteoporosis Patient Network (80% aged >60 years; 75% had a previous fracture) and 34 clinicians comprising physicians, nurses, physiotherapists, and pharmacists. We identified 3 categories of functionalities to be included in the app: support resources, diary, and educational materials (Multimedia Appendices 2 and 3 provide the full list of requirements). The support resources guide the users on how to use the app and when and where to seek medical assistance after fractures. The diary functionalities record the pain levels and medication intake of the users. The educational materials provide information on pain management, healing and recovery, mobility, and psychological well-being. We then produced a low-fidelity paper prototype that met all functional and accessibility requirements (Figure 2).

**Figure 3:**
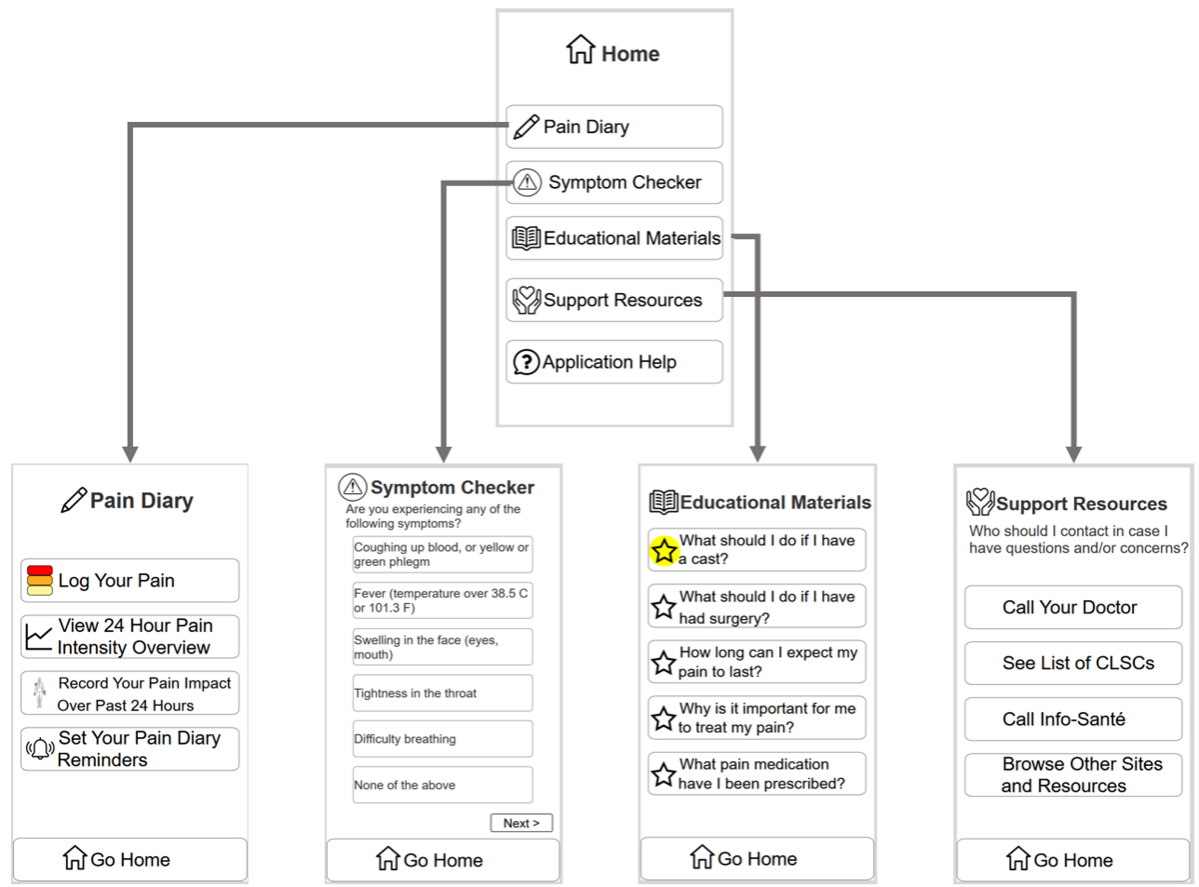
Digital low-fidelity prototype, showing the home page, pain diary page, symptom checker page, educational materials page, and support resources page.

In preparation for the next phase of the study, we converted the paper prototype into a digital low-fidelity prototype. As the prototype was converted, we discussed the designs in the group and simplified or recategorized some functionalities to reduce the number of features for ease of use. This reorganization was also performed in an attempt to further flatten the menu structure of the app. For example, the “mood diary” and “reports” in the paper prototype (Figure 2) were merged with the “pain diary” in the first iteration of the complete digital low-fidelity prototype (Figure 3).

### Phase 2—Participatory Design Workshops

#### Overview

**Figure 4:**
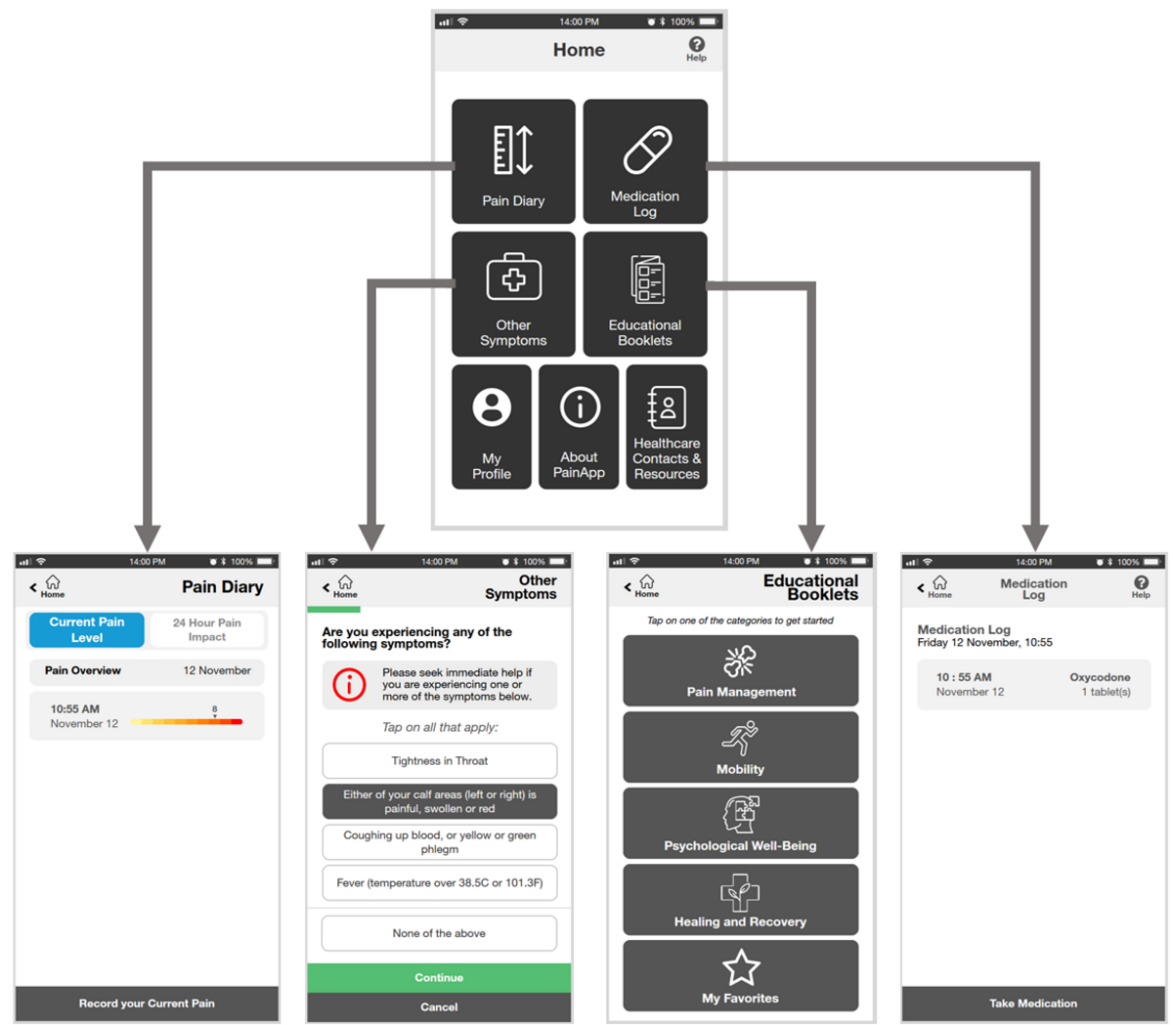
Digital medium-fidelity prototype, showing the home page, pain diary page, symptom checker page, educational materials page, and medication log page.

We conducted 4 participatory design workshops with 6 participants (4/6, 67% women; 2/6, 33% men; mean age 76.7, SD 9.5 years) from March 2021 to July 2021; all the participants had experienced a fracture after the age of 40 years. In the resulting codebook (Table 1), four overarching categories emerged from the thematic analysis of the workshops: (1) feedback on the user interface and usability, (2) request for additional app functionalities, (3) feedback on medical guides and educational materials, and (4) suggestions for additional medical educational materials. We do not provide quantitative information such as counting the number of times information (ie, words, terms, and sentences) was mentioned, as the goal of our study was to reveal unexpected findings in an inductive data collection and analysis process, and counting such information would not have theoretical value [40]. We used the feedback from the first 3 workshops to design a medium-fidelity prototype (Figure 4) in which we added and modified the content and features, refined the visual design, and added interactivity. In the fourth workshop, participants reviewed our medium-fidelity prototype, validated the changes, and provided feedback to further refine our medium-fidelity prototype.

**Table 1:** Codebook resulting from the thematic analysis.

Code		Design workshop representative quotes
**1. Feedback on app user interface and usability**		
	1.1 Navigation between pages should be intuitive	“The problem when you’re a novice is getting back to where you started. I can’t tell you how many times I’ve had to log out and start all over again.” [Participant 03]
	1.2 Pain scale color scheme should match the level of pain	“[The yellow to red] is a recognized sequence.” [Participant 05]
	1.3 Screens should not be overcrowded	“Because I don’t want to make the screen any busier.” [Participant 02]
	1.4 Users should be provided instructions on how to interact with the app	“You need to be told to click because we just don’t automatically click.” [Participant 03]
	1.5 The app should limit the number of steps the user has to go through to complete a task	“It could be as simple as a button you push when you take your medication.” [Participant 01]
	1.6 Urgent medical information should be highly visible and easily accessible	“If you click on it, it [could] take you over to more information on why a red flag symptom is important to act on immediately.” [Participant 01]
	1.7 The app should have pleasing visual graphics	“Are you going to add any graphics [...] where you put all that whitespace?” [Participant 01]
**2. Requests for additional app functionalities**		
	2.1 Medication tracker	“I found it very helpful to make a list of when I took the medication because you think you’ll remember, but you don’t.” [Participant 02]
	2.2 Logging pain reminder	“But could it also prompt you with, you know, the time for your next recommended time for your [...] next evaluation of pain?” [Participant 01]
	2.3 Warnings for medication overdosage	“Will [the application] tell you to make sure that you don’t take any more narcotics if you have extreme pain? Because that will be a very bad thing for you.” [Participant 04]
	2.4 Data visualization of pain entries	“I’m thinking of the Environment Canada weather app. What we could do is have the pain scale on the vertical axis and the times across the bottom.” [Participant 01]
	2.5 Categorizing contacts in the address book	“Your contact afterwards may not be your family doctor. So [you may need more than] only one health professional contact that you can put in the app.” [Participant 02]
**3. Feedback on app medical guides and educational materials**		
	3.1 App provides helpful information on red flag symptoms	“I like the idea of red flag symptoms because those are the things that you should address immediately.” [Participant 01]
	3.2 App acts as a reinforcement tool for the information given at the hospital	“So this, to me, is a reinforcement of stuff you’ve already been told.” [Participant 02]
	3.3 App provides helpful information on pain management strategies	“He was given no advice on pain [management], so I think this is excellent.” [Participant 06]
	3.4 App allows for better communication between the user and their health care provider	“It’s difficult for them to share what it has been like for the past 6 weeks or whatever, and the tool will help them communicate that to their doctor.” [Participant 02]
	3.5 The app should use layman’s terms	“Tightness in the throat might be better explained as difficulty in swallowing.” [Participant 05]
	3.6 The app should use inclusive language	“The ‘back on your feet’—it’s just sensitive to people, let’s say they’re in a wheelchair, right?” [Participant 02]
**4. Suggestions for additional medical educational materials**		
	4.1 The app should provide information about do’s and do not’s after injury	“So, there’s really some do’s and don’t’s that you should know when you leave the hospital.” [Participant 02]
	4.2 The app should guide users when and where to seek medical assistance	“Is there going to be a point where [the application] says, at what stage should you contact your doctor to discuss something else to help better alleviate your pain?” [Participant 02]
	4.3 The app should have information about early mobility	“We’re concerned about how to manage not only the pain but the movement.” [Participant 03]
	4.4 The app should contain links to external websites for tailored information about various injuries	“Is there a website or something that you could direct people to so that they could get [...] information [...] for immediate aftercare of a spinal fracture?” [Participant 03]
	4.5 The app should provide personalized medical content	“With a vertebral fracture, you’re handed your pain medication, and you were out in the cold. So some kind of very specific suggestions concerning coping with a vertebral fracture [would be nice to have in the application].” [Participant 03]

#### Feedback on User Interface and Usability

Participants commented on the appearance and usability of the app. The participants liked the color scheme used for the pain scales, which had a spectrum from yellow (toward less pain) to red (toward more pain) that was intuitive. They also liked having an option to easily return to the home page via a large icon on the header. The participants wanted urgent medical information, such as warnings for life-threatening symptoms, to be easily discernible from the rest of the content to create a sense of urgency. Participants wanted explicit step-by-step instructions on how to interact with the app, such as “Please tap here to continue” and an explanation of the rationale behind each functionality. They pointed out usability issues, such as crowded screens, unintuitive navigation, and poor content discoverability.

We resolved any usability issues pointed out by the participants as our priority because we consider these to be impediments to using the app effectively. These changes were made after each workshop and included (1) decreasing the complexity of the app by removing unnecessary screens; (2) making important information more salient by bolding it with high-contrast colors (eg, red); (3) providing tutorials, in the form of pop-ups, with instructions and explanations on functionality use and benefits; and (4) adding more icons and illustrations to aid navigation and make the app aesthetically pleasing.

#### Requests for Additional App Functionalities

The participants proposed additional functionalities to be included in the app that would be helpful for pain management. Namely, the participants felt that reminders to log their pain would motivate them to use the app; they wanted to visualize their pain levels on a chart as time progresses, as this would facilitate at-a-glance monitoring; participants indicated having difficulty keeping track of their medication intake owing to high levels of pain and the brain fog caused by pain medication; and they requested functionalities to keep track of their intake and to warn them if they attempted to take medications at a higher frequency than prescribed. Although participants appreciated the contact book of health care providers in the app, they indicated that it would be useful to be able to categorize contacts; this function will help them and their caretakers to quickly identify who to call.

Consistent with the demands of the participants, we designed and implemented these functionalities after the first 3 workshops. These included (1) reminders to log pain entries; (2) a medication diary, which included warnings if the user attempted to record medication intake too often; and (3) the ability to categorize contacts as “Family Doctor,” “Pharmacist,” “Occupational or Physical Therapist,” “Homecare,” “Clinic,” “Hospital,” and “Other” when saving a contact in the app. We then presented and validated these new functionalities in the fourth workshop, which were all well received.

#### Feedback on Medical Guides and Educational Materials

Participants responded favorably to medical content on acute pain management; they felt they were not given adequate information after hospital discharge and that the mHealth app would provide useful information. Participants also saw the value of the “pain diary,” as it would allow them to monitor their pain levels and communicate their pain journey more effectively with their health care providers. Participants critiqued some of the wordings of content in the app. In response to their comments, we simplified medical jargon into layman’s language. We also changed the wording to offer a more inclusive language.

#### Suggestions for Additional Medical Content

Although the participants perceived the medical content on acute pain management as satisfactory, they indicated that the app lacked recovery and early mobility information. Furthermore, 2 participants stressed the importance of including information about early mobility when recovering from injuries such as hip or vertebral fractures. Participants also wanted practical advice regarding when to seek medical assistance, such as whether they should contact their health care provider if their severe pain does not abate.

Suggestions for additional medical content were reviewed by the clinician-researcher in our team and then added to the app. These included (1) specific practical advice on “dos” and “don’ts” for different types of injuries to supplement general advice, (2) an alert encouraging users to seek medical advice when their pain is uncontrolled (3 consecutive pain scores rated at ≥7), (3) resources leading to external links (such as Osteoporosis Canada) for information outside the scope of this app, and (4) information on early mobility.

### Phase 3—Usability Testing

#### Overview

**Figure 5:**
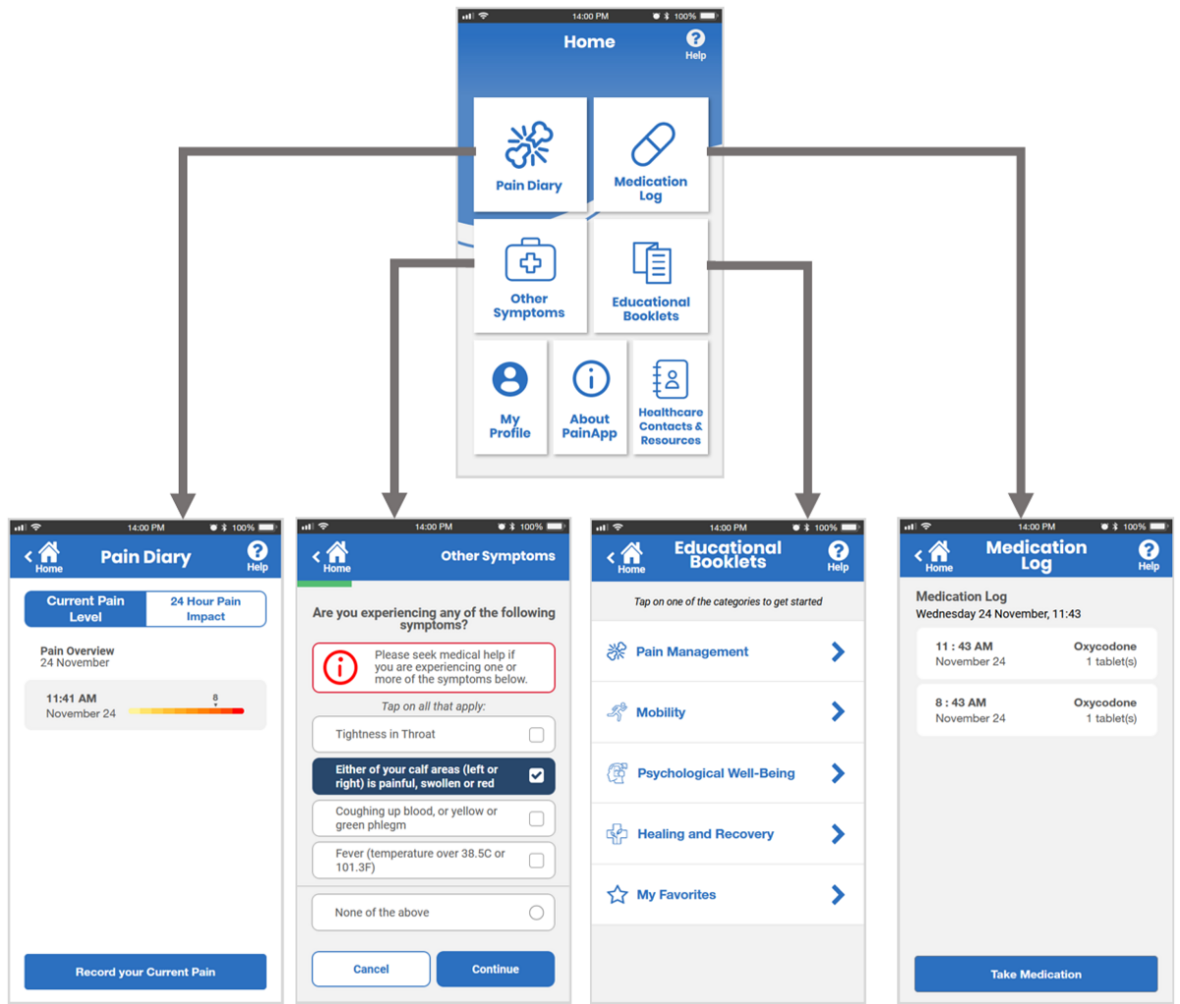
Digital high-fidelity prototype, showing the home page, pain diary page, symptom checker page, educational materials page, and medication log page.

We evaluated the usability of our medium-fidelity prototype with 10 participants (7/10, 70% women; 3/10, 30% men; mean age 68.6, SD 4.12 years) in October 2021. All the participants had experienced at least one fracture after the age of 40 years. We analyzed the results of the usability test sessions to design the high-fidelity prototype (Figure 5) and finalized the content, features, and visual design required in the preparation for professional app development.

#### Overall Impressions of the App

Participants’ overall impressions of the app were positive, with many indicating that they would have used it if it were available at the time of their injury.

##### Ease of Use

All the participants found the app easy to use, including even those who stated that they usually found new technology difficult to use:

Even for someone like me, I found it easy. But I would have had my son or daughter to help me if I had any questions.

Participant 15

##### Personalization

Each person’s experience with fractures was different. Participants who had minimal pain were interested in educating themselves on their injuries and steps toward recovery rather than addressing pain:

I didn’t need medication. I had a little pain. [...] So, for me, general information would have been good because there were many things I didn’t know.

Participant 09

Participants who experienced more pain after their fracture were interested in app functionalities directly related to managing acute pain, such as recording the time and dosage of pain medication intake:

I think the very first thing I would be interested in is the medication log. Because seeing how much I need to take day to day, I think, is crucial.

Participant 15

##### Communication Facilitator

Participants perceived value in using the app as a communication tool when visiting their health care providers. Participants thought the pain diary could act as a memory aid to help them recall their overall health and issues they face:

I think it’s great because you go to the doctor and you sort of forget to say things [...] or you forget to ask some questions.

Participant 14

#### Types of Difficulties During Task Performance

##### Overview

Although the overall participants’ experience with the prototype was positive, the usability tests revealed problems with the user interface and areas for improvement. We addressed the problems encountered by the participants during the usability tests in the final version of our prototype. Multimedia Appendix 4 provides a full list of the identified and resolved difficulties.

##### Identifying Interactable Elements

A few participants failed to notice a button or an input field or mistook noninteractable elements for buttons, such as textboxes with a border.

##### Navigation

Lack of navigation affordance caused issues among some participants. The participants failed to realize that they could scroll on pages to access information beyond the screen frame. The hub-and-spoke navigation pattern worked well, as we did not notice participants struggle with returning to the home page or accessing different parts of the app. Most of the navigation problems were related to the nomenclature of the items on the main menu and some buttons, as participants noted that the name of the item or the button was not indicative of the feature or function.

##### Data Entry Interactions

In some instances, participants either failed to enter the expected information or completely missed inputting information. Incorrect field completion and omission of input may have been because of the high cognitive effort required to carefully read questions or confusion caused by the question’s phrasing.

##### Lack of Error Recovery

Some participants inadvertently skipped the onboarding process because they were unsure if they were required to continue and pressed the “skip” button.

#### Feedback Provided During Task Performance

In addition to the identified usability problems, the participants provided feedback on aspects of the app that were confusing or cumbersome.

##### Interpretation of Information

As the participants navigated to access different functionalities of our app, tutorials appeared as pop-ups on how to use the functionality. A few participants skipped some of the tutorials and noted that they were too long. In addition, some participants indicated that they would have liked the tutorials to explain the reasoning and benefits of using a certain feature. For example, they wanted to know how long they should be using the pain diary and how it would help them manage their pain.

##### High Loads of Cognitive Effort Required

Some participants indicated that they would not use the Brief Pain Inventory—short form part of the “pain diary” regularly, as they thought it took too long to complete. The Brief Pain Inventory—short form is a 10-item, validated and self-administered questionnaire to assess pain and its impact on daily functioning [41]. In addition, most participants had trouble remembering which medications and dosages they were prescribed when the app asked them to input their prescriptions. Some questions in the app were poorly formulated; for example, participants expressed confusion over a question in the profile setup, which asked, “Were you prescribed or recommended to take acetaminophen for your pain?” As the possible answers were either “Yes” or “No,” participants found it hard to answer the double-barreled question as they thought they were being asked 2 questions in 1.

## Discussion

### Principal Findings

This study demonstrated how we designed, developed, and evaluated an mHealth app prototype to empower older adults to self-manage their acute pain after a fracture using a 4-step HCD approach: (1) definition of context of use, (2) identification of user requirements, (3) production of design solutions, and (4) evaluation of design solutions to design our mHealth app [42]. Our multidisciplinary team is one of the strengths of our study, as experts from various fields provided input in the design of the app. Inputs from a physician and from pharmacists were considered, as they ensured the accuracy of our medical content. Another strength of our study is the involvement of older adults, which allowed us to gain a deeper understanding of their needs and frustrations when using mHealth technologies to manage their acute pain.

It is widely believed that older adults are often fearful and unwilling to try new technologies [43,44]; our findings challenge this stereotype, as the participatory design workshops and usability tests revealed a need and enthusiasm for an acute pain management mHealth app. Many participants from our design workshops and usability tests expressed their willingness to use mHealth technology to assist in their pain and injury management journey.

In line with other studies on mHealth tools, we also found that people are looking for clear, concise, and personalized health content [45-47]. Methods of managing pain and recovery may differ based on the type of injury, and a one-size-fits-all approach will rarely meet the users’ needs; health content that is relatable to one’s particular case is often perceived as more beneficial [48]. As stated in the literature [49,50], we found that it is important to provide clear instructions on how to use the app for older adults. Older adults often view the use of technology as a series of steps or procedures and are less inclined toward trial-and-error learning styles owing to the fear of “breaking something” [51]. In addition, our participants highlighted the importance of understanding the benefits of using the app; previous studies also echoed the importance of communicating the benefits of using the proposed technology to older adults [52].

Through design workshops, we found that participants who had sustained skeletal fractures considered mobility as important as pain management. We had originally emphasized pain management in the initial phase of the study but quickly pivoted to include more educational materials on mobility in the second phase. The sentiment of mobility being an important topic was echoed by the participants in the usability tests in the third phase, who were pleased to see a large number of educational materials related to mobility.

We established evidence-based design requirements from needs assessment studies, accessibility design guidelines for older adults from the literature, and participants’ feedback on our design iterations. These efforts contributed to our final mHealth prototype design that all the participants perceived as useful and easy to use. Although the overall results of our usability tests were positive, we uncovered some design problems. Similar to previous studies, scrolling [53,54], identifying buttons to trigger an action [54-56], and interacting with nonactionable targets [32] were the most common problems encountered in our evaluations. Therefore, we recommend that designers be mindful of these potential difficulties when designing mHealth apps for older adults.

### Limitations

Our study is not without limitations. First, our studies were conducted remotely owing to the COVID-19 pandemic restrictions, which may have affected the participants’ interactions with the prototype. We used only an emulator on a desktop computer to test our medium-fidelity mHealth prototype; thus, it is possible that some usability problems were not detected. In the future, the prototype should be tested on a smartphone to approximate real use conditions. Second, convenience sampling may have biased our results, as we recruited participants who had participated in previous studies on bone health or who had been evaluated in orthopedic clinics at our center. Design workshop participants were well aware of osteoporosis and its negative impacts and may have had prior knowledge related to injury management. Nevertheless, we believe that they represent the population of patients with skeletal fragility who might use such a tool for pain management following a fracture. In the future, the prototype should be tested with participants with limited or no knowledge of managing an injury. Third, the protocol for the usability tests required participants to have access to Zoom. Consequently, these participants likely had higher technological literacy than those who would never have used videoconferencing tools. In the future, this app should be evaluated by participants with low technological literacy, and the design should be modified accordingly.

### Conclusions

Our prototype results from the needs assessment surveys and the insights provided in co-design workshops and usability tests, with content developed in partnership with practicing health care professionals. Our prototype is promising, as the usability test results indicate that the prototype was easy to use for the older adults who participated in this study and contained useful materials. Researchers aiming to develop mHealth technologies would benefit from an HCD approach, as this method promotes the establishment of evidence-based requirements and eliminates potential frustrations early in the design process through continuous evaluation of iterations. In future studies, we plan to professionally develop this prototype on mobile devices and evaluate the impact of the app’s use on patient health outcomes through clinical trials and longitudinal studies.

## Appendix

Multimedia Appendix 1 Usability test interview plan.

Multimedia Appendix 2 Initial design requirements identified from prior surveys with members of the Canadian Osteoporosis Patient Network and health care providers.

Multimedia Appendix 3 Usability and interface accessibility requirements identified from the literature.

Multimedia Appendix 4 Problems and feedback encountered by participants during usability testing and our proposed fixes.

## Abbreviations

HCD: human-centered design
mHealth: mobile health

## Data Availability

Data sets generated and analyzed during this study are available from the corresponding author upon request.
