# Supplementary material for: Mobile App Prototype in Older Adults for Postfracture Acute Pain Management: User-Centered Design Approach
Source: JMIR Aging. 2022 Oct 17;5(4):e37772. doi: 10.2196/37772 (PMC9635443; doi:10.2196/37772)
Supplement: Multimedia Appendix 1 [file aging_v5i4e37772_app1.docx]

## Multimedia Appendix 1

Usability test interview plan

**Tasks**

1. Create an account: Please think back to the time when you injured yourself. You’ve just downloaded the application. Please create a new profile.

2. Log a pain entry: Please think back to the time when you injured yourself, maybe a few days after you fractured your bone(s). How would you keep track of your pain using the application?

3. Log medication: Let’s say you’ve just taken 2 tablets of Tylenol for your pain. How would you keep track of your medication intake using the application?

4. Educational booklets: You would like to find out what you can do to help yourself recover faster from your injuries. Please find information on this topic, and let me know once you are satisfied with what you have learned.

5. Symptom checker: Let’s say you are experiencing some swelling in your face and are feeling a little nauseous. You are not sure if this is normal. How would you find out if you need to seek medical assistance or not?

6. Add a contact: You want to keep a list of phone numbers of your healthcare team, such as your family physician, in case you need to contact them quickly. Please record your family physician’s phone number in the application.

7. Update prescription: [Direct participant back to Medication Log]. You just took a tablet of X, but you don’t see it in your choices. Do you remember at the beginning, when you were asked if you were prescribed X? Let’s say that you made a mistake and were, in fact, prescribed Y. How would you fix this mistake?

8. Find the nearest pharmacy: Let’s say you are in another town, and you need to buy some medication. How would you find out where to buy medication using the application?

9. Change language: Please change the language of the application to French.

10. Log a 24-Hr Pain Assessment (BPI-sf): Please fill in a 24-Hr Pain Assessment.

**Closing Questions**

1. What was your overall impression of the application?

2. If you had to change one thing about the app, what would it be?
