# Supplementary material for: Mobile App Prototype in Older Adults for Postfracture Acute Pain Management: User-Centered Design Approach
Source: JMIR Aging. 2022 Oct 17;5(4):e37772. doi: 10.2196/37772 (PMC9635443; doi:10.2196/37772)
Supplement: Multimedia Appendix 2 [file aging_v5i4e37772_app2.docx]

## Multimedia Appendix 2

Initial design requirements identified from prior surveys with members of the Canadian Osteoporosis Patient Network and healthcare providers.

| Category | Requirement |
| --- | --- |
| **Support Resources** |  |
|  | The application needs to include links to medical support (pharmacy, ER, walk-in clinics) along with their contact information such as phone number, website, email and postal address. |
|  | The application needs to include guides for patients on when to seek emergency care. |
|  | The application needs to include links to community support resources along with their contact information such as phone number, website, email and postal address. |
|  | The application needs to include links to rapid IT support. |
|  | The application needs to include a help page with advice on how to use the application. |
| **Diary** |  |
|  | The application needs to support pain tracking, including the date, time, localization and intensity (1-10) of the pain. |
|  | The application needs to provide a view to visualize pain progress. |
|  | The application needs to provide a historical log of pain for clinicians. |
|  | The application needs to support medication logging, including the time taken, dosage, type of medication and side effects. |
|  | The application needs to send notifications and reminders to take scheduled medications. |
|  | The application needs an easily accessible list of all medications the patient is taking. |
|  | The application needs to have a calendar for upcoming medical appointments. |
| **Educational Materials** |  |
|  | The application must provide educational materials on mobility (assistive devices, cast, exercise, prevention of falls and fractures). |
|  | The application must provide educational materials on pain management (medications, non-pharmacological pain management, fracture specific materials, expectations & timelines of pain duration). |
|  | The application must provide educational materials on psychological well-being. |
|  | The application must provide educational materials on healing and recovery. |
