# Supplementary material for: Mobile App Prototype in Older Adults for Postfracture Acute Pain Management: User-Centered Design Approach
Source: JMIR Aging. 2022 Oct 17;5(4):e37772. doi: 10.2196/37772 (PMC9635443; doi:10.2196/37772)
Supplement: Multimedia Appendix 3 [file aging_v5i4e37772_app3.docx]

## Multimedia Appendix 3

Usability and interface accessibility requirements identified from the literature.

| Category | Requirement |
| --- | --- |
| **Usability** |  |
|  | The application should not require gestures (pinch, spread, double tap, one- vs two- vs three fingers drag). |
|  | The application needs to have consistent navigation patterns (the same menu throughout). |
|  | The application needs to use a hub-and-spoke navigation pattern. |
|  | The application needs to leave menus open. |
|  | The application needs to have a consistent layout through pages. |
|  | The content must be concise and understandable (use layman’s terms). |
|  | The content must be accompanied by concrete examples (medication, therapies, what to expect). |
| **User Interface** |  |
|  | The application needs to have a solid, off-white background for vision sensitivity to glare. |
|  | The application needs to have labels for icons and symbols. |
|  | The application needs to make notification signals large enough and with distinct coloring to be noticed. |
|  | The application needs to provide cues in the interface about the user's recent action (recognizing where the users are is easier than recalling what they have done). |
|  | The application needs to have sufficient contrast between text and background. |
|  | The application’s font sizes should be larger than 12-14 points. |
|  | The application’s font families should be simple. |
|  | The application should have fonts of a regular thickness and avoid thin text. |
|  | The application should be mixed case and avoid all caps. |
|  | The application needs to have buttons positioned following commonly known app design patterns. For example, a search bar should be in the upper right-hand corner. |
|  | The application needs buttons that are at least 16.5mm diagonally. |
|  | The application needs to support margins between buttons. |
