# Supplementary material for: Mobile App Prototype in Older Adults for Postfracture Acute Pain Management: User-Centered Design Approach
Source: JMIR Aging. 2022 Oct 17;5(4):e37772. doi: 10.2196/37772 (PMC9635443; doi:10.2196/37772)
Supplement: Multimedia Appendix 4 [file aging_v5i4e37772_app4.docx]

## Multimedia Appendix 4

Problems and feedback encountered by participants during usability testing and our proposed fixes.

| Category of Problem (Error or Feedback) | Issue(s) | Fix(es) |
| --- | --- | --- |
| **Identifying interactive elements (Error)** |  |  |
|  | Did not notice Call to Action buttons | Used bright colors with high contrast from the background for all buttons |
|  | Did not notice the input field | Used borders with high contrast from the background for input fields |
|  | Tried to tap on areas that were not interactive (text) | Differentiate interactive from non-interactive elements (see two previous rows) |
|  | Were unable to change the time of medication intake due to controls being too small | Made areas of touch large (at least 100 by 50 pixels) |
| **Navigation (Error)** |  |  |
|  | Did not notice the page could be scrolled | Added a snackbar (brief message at the bottom of the screen) to indicate when users can scroll |
|  | Confused about the nomenclature of main menu items | Moved content in the category that better matches the users’ mental model (e.g. moved “Language” from “About the App” to “My Profile) |
|  | Labels on the button were not indicative of function | Specified button labels (e.g. “Go Back to Categories” instead of “Go Back”) |
| **Data entry interactions (Error)** |  |  |
|  | Incorrectly entered full name in the first name field | Clarified instructions in the question (“What is your first name?”) and labelled the input field (“First Name”) |
|  | Incorrectly entered full dosage (e.g. 2 x 500mg tablets) in the individual tablet dosage field | Clarified instructions to enter the dosage for 1 tablet and added error validation for dosage values that are higher than what is normally prescribed |
|  | Did not fill in all required fields | Added warning or error message to remind users to fill in all required fields |
| **Error recovery (Error)** |  |  |
|  | Skipped the initial onboarding process by tapping Skip | Added a dialog box to confirm if the user wishes to skip to facilitate error recovery |
| **Interpreting information (Feedback)** |  |  |
|  | Did not fully understand the benefits of Pain Diary feature | Revised tutorial for the pain diary |
|  | Had trouble understanding or did not read the tutorial due to information overload | Shortened the contents in the tutorials for each feature |
|  | Did not understand why they were asked to select painful areas on the body (on BPI-sf) | Clarified instructions on how to answer the BPI-sf item |
| **Requiring High Cognitive effort (Feedback)** |  |  |
|  | The BPI-sf questionnaire was long | Shortened the BPI-sf questionnaire |
|  | Had trouble remembering medication dosage | Provided pictorial memory aids |
|  | Choice of answers did not match question being asked in the profile setup when prompted to enter prescription | Revised question formulation |
